# Characteristics of Obese Patients with Acute Hypercapnia Respiratory Failure Admitted in the Department of Pneumology: An Observational Study of a North African Population

**DOI:** 10.1155/2022/5398460

**Published:** 2022-02-17

**Authors:** Sameh Msaad, Rahma Gargouri, Amina Kotti, Nesrine Kallel, Amel Saidane, Yassine Jmal, Wajdi Ketata, Nadia Moussa, Amine Bahloul, Samy Kammoun, Jihene Jdidi

**Affiliations:** ^1^Faculty of Medicine, University of Sfax, Tunisia; ^2^Department of Respiratory and Sleep Medicine, Hedi Chaker University Hospital, Sfax, Tunisia; ^3^Department of Cardiology, Hedi Chaker University Hospital, Sfax, Tunisia; ^4^Department of Preventive Medicine, Hedi Chaker University Hospital, Sfax, Tunisia

## Abstract

**Background:**

Acute hypercapnic respiratory failure (AHRF) is a common life-threatening event in patients with obesity hypoventilation syndrome (OHS).

**Objectives:**

To study the clinical pattern, noninvasive ventilatory support, as well as the short- and long-term outcomes of patients with OHS admitted in a ward because of AHRF.

**Methods:**

We conducted a retrospective cohort study including all adults with OHS aged ≥ 18 − year − old, admitted in a 90-bed-ward for AHRF.

**Results:**

A total of 44 patients were included. Fifteen (34.1%) and 29 (65.9%) patients were diagnosed with malignant OHS (mOHS) and nonmalignant OHS (non-mOHS), respectively, while 36 (81.8%) had coexisting obstructive sleep apnea hypopnea syndrome (OSAHS). Patients with mOHS had a significantly higher rate of heart failure (100% vs. 31%; *p* < 0.001), chronic renal insufficiency (CRI) (73.3% vs. 41.4%; *p* = 0.04), and dyslipidemia (66.7% vs. 34.5%; *p* = 0.04) than those with non-mOHS. The mean forced vital capacity (FVC) in our patients was of 59.5% ± 18.5 of the predicted value, lower than what is usually reported in stable patients with OHS. At hospital admission, more than two-thirds (*n* = 34, 77.3%) were misdiagnosed as having asthma exacerbation (*n* = 4, 4.9.1%), chronic obstructive pulmonary disease (COPD) exacerbation (*n* = 12, 27.3%) and/or heart failure (*n* = 29, 65.9%). Acute pulmonary oedema (ACPE) (*n* = 16, 36.4%) and acute viral bronchitis (*n* = 12, 27.3%) were the main identified causal factors, while no cause could be determined in 5 (11.4%) patients. Noninvasive positive pressure ventilation (NIPPV) using bilevel positive airway pressure (BIPAP) was very highly effective to treat AHRF, with only 2.27% of patients failing the modality. Median overall duration of ventilation was 9 hours per day (1.3–20) and was significantly longer in patients with mOHS than in those with non-mOHS (10 [6–18] vs. 8 [1.3–20], respectively; *p* = 0.01). Forty two of the forty-three patients discharged alive were treated with BIPAP or continuous positive airway pressure (CPAP) in 26 and 16 patients, respectively. The probability of survival was 90% at 12 months, while the probability of readmission for a new episode of AHRF was 56% at 6 months and 22% at 12 months, respectively.

**Conclusion:**

AHRF in OHS patients is a life-threatening event which can be successfully and safely treated with BIPAP, with a low long-term mortality even in patients with mOHS.

## 1. Introduction

Over the recent decades, the prevalence of obesity in North African countries has greatly increased due to the rapid epidemiological transition and dietary behaviour changes [1]. Tunisia is one of these countries, and today features a high prevalence of obesity that has almost tripled over the last three decades, according to the latest statistics of the World Health Organization (WHO). It has increased from 8.7% in 1980 (Statistiques [2]) to 28% in 2010 among adults > 30 years, which means that over three million Tunisian adults are obese [3]. As observed in the majority of countries in the region, obesity is more common in women than men, with close to one third of Tunisian women reported to be obese [4].

With this increasing obesity rate, the Tunisian population has experienced a major rise in the prevalence of many obesity-related diseases, in particular, type 2 diabetes, cardiovascular diseases, and the metabolic syndrome which today affect nearly a third of Tunisian adults [5]. The accrual in obesity is also likely to lead to an increase in sleep-related breathing disorders including obesity hypoventilation syndrome (OHS) and obstructive sleep apnea/hypopnea syndrome (OSAHS).

OHS, previously known as Pickwickian syndrome, is defined as the appearance of awake hypercapnia (arterial partial pressure of carbon dioxide [PaCO_2_] > 45 mmHg) in the obese patient (body mass index [BMI] > 30 kg/m^2^) in the absence of other known causes of alveolar hypoventilation such as lung or neuromuscular diseases [6]. Most patients with OHS have coexisting OSAHS. Though there is still insufficient epidemiological data, current estimations suggest that the prevalence of OHS is less than 1% of the general population [7]. It increases significantly as the prevalence of obesity increases, with a reported prevalence ranging from 10% to 20% in outpatients presenting with suspected sleep-disordered breathing (SDB), and 30% in hospitalized obese patients [6]. To our knowledge, no data about the prevalence of OHS in North Africa either in the general population or in patients with OSAHS are available.

Comorbidities and complications are common in patients with OHS, which leads to high hospitalization rates, more health care expenses, lower quality of life, with higher morbidity, and mortality. OHS may cause chronic complicacies such as pulmonary hypertension (PHT), right heart failure, and acute hypoxemic or most commonly hypercapnic respiratory failure (AHRF).

Despite its high prevalence and significant morbidity and mortality, OHS remains largely under recognized and usually discovered at an advanced stage when AHRF occurs ([8, 9]. The number of patients admitted to the intensive care unit (ICU) with AHRF due to previously undiagnosed OHS is also increasing [10]. However, among these patients, only 30% of OHS patients receive a correct diagnosis when admitted with AHRF, while up to 75% are misdiagnosed as having chronic obstructive pulmonary disease (COPD) or asthma [11]. Consequently, many patients do not receive the appropriate treatment, which may result in a higher risk for readmission for new episodes of AHRF, an additional cost, and increased morbimortality [12] [13].

Noninvasive positive pressure ventilation (NIPPV) using bilevel positive airway pressure (BiPAP) or continuous positive airway pressure (CPAP) is recommended as the primary management option for stable ambulatory patients diagnosed with OHS [14]. Moreover, BIPAP is being increasingly used during AHRF in patients having probable or confirmed OHS, with acceptable rates of success [15]. So far, there has been no published consensus either on indications or on the protocol for the proper application of BIPAP in AHRF complicating OHS.

We retrospectively studied a cohort of patients with OHS admitted in a ward with AHRF to examine their clinical pattern, their noninvasive ventilatory support, and their short- and long-term outcomes.

## 2. Materials and Methods

### 2.1. Study Design

We conducted a retrospective observational cohort study in the respiratory disease department of the Hedi Chaker University Hospital in Sfax, Tunisia. Around 2000 patients a year are admitted at this department which has a capacity of 90 beds.

### 2.2. Population

#### 2.2.1. Inclusion Criteria

All obese adults aged ≥ 18-year-old, admitted in our department between 1 January 2012 and December 2019 for AHRF, were consecutively enrolled in this study. According to the International Classification of Diseases, Tenth Revision, Clinical Modification (ICD-10-CM), patients without chronic respiratory failure (CRF) were diagnosed with acute hypoxemic respiratory failure if the PaO_2_ was <60 mmHg (SpO_2_ < 91%) on room air, or the PaO_2_/fraction inspired oxygen (FiO_2_) (P/F) ratio was <300. In patients with CRF, a PaO_2_ < 60 mmHg on their usual supplemental oxygen flow rate, or a 10 mmHg decrease in baseline PaO_2_ (if known) indicated acute hypoxemic respiratory failure. AHRF was defined as an arterial pH less than 7.35, associated with a PaCO_2_ > 45 mmHg, or an increase by 10 mmHg in the baseline PaCO_2_ (if known) [16].

#### 2.2.2. Noninclusion Criteria

The noninclusion criteria were a smoking history of 20 pack-years or more, a diagnosis of COPD with forced expiratory volume first second (FEV1) < 50% predicted, neuromuscular disease, chest wall disease, kyphoscoliosis, diaphragmatic paralysis, restrictive pulmonary disease, idiopathic and postcapillary PHT, or other known reasons for hypoventilation disorders such as sleep-related hypoventilation due to a medication or substance and idiopathic central alveolar hypoventilation. Malignant OHS (mOHS) was defined as a severe form of OHS characterized by a BMI > 40 kg/m^2^, metabolic syndrome (central obesity, hypertension, hyperlipidemia, and insulin resistance), and multiorgan system dysfunction related to obesity [10] [17]. BMI was calculated by dividing weight (in kilograms) by squared height (in meters). Obesity was defined as a BMI equal or greater than 30 kg/m^2^. Three ranges of BMIs were used to assess the severity of obesity: class I obesity if BMI was 30.0 to 34.9 kg/m^2^, class II obesity, if BMI was 35.0 to 39.9 kg/m^2^, and class III or morbid obesity, if BMI ≥ 40 kg/m^2^ [18].

### 2.3. Data Collection

For each patient, only data at the first admission for AHRF to our department during the period of the study were taken into account. Clinical data from the first day of admission were recorded. Regarding data relative to para-clinic investigations, we noted the most recent tests performed before admission to our department (within the last 2 years). If not available, tests obtained when patients had stabilized (after hospital discharge) were considered.

#### 2.3.1. Demographic Data and Clinical History

Data collected during hospital stay included gender, age, smoking status, commodities, medical history, clinical presentation, and conditions precipitating respiratory failure. Previous ICU stay (s) and previous treatment with NIPPV at home were also reported. Data relative to in-hospital therapeutic management included pharmacological treatment, as well as modalities, duration, and effectiveness of NIPPV. NIPPV was considered successful when intubation was avoided and the patient discharged alive. Failure of NIPPV therapy occurred when, despite optimal support, worsening of respiratory distress and gazometric disturbance was observed leading to intubation, transfer to ICU, or death [19]. Time to discharge from our department or to in-hospital death was registered, as well as readmissions for new episodes of AHRF and death after discharge.

#### 2.3.2. Laboratory Findings

The following available data were collected: serum glucose level, complete blood count, blood urea nitrogen (BUN) levels, serum creatinine levels, alanine transaminase (ALT), aspartate transaminase (AST) levels, C-reactive protein (CRP) levels, serum N-terminal pro-brain natriuretic peptide (NT-pro-BNP) level, serum troponin level, and thyroid-stimulating hormone (TSH). Chronic renal insufficiency (CRI) was defined as a baseline serum creatinine clearance < 90 ml/min, and hepatic cytolysis as an ALT level > 40 UI/L. CRP > 0.6 mg/dL was considered abnormal [10]. Hypothyroidism was defined as a TSH level > 4.50 mU/L [20]. Regarding NT-ProBNP level, the following upper reference limits (URLs) were applied: 450 ng/L for <50 years, 900 ng/L for 50–75 years, and 1800 ng/L for >75 years [21]. Arterial blood gas (ABG) data were recorded at admission, at discharge, and three months after discharge.

#### 2.3.3. Respiratory Function Test

The results including FEV1, forced vital capacity (FVC), and FEV1/FVC were compared with local spirometric norms calculated from the patient's height, weight, and age using equations from the European Respiratory Society (ERS) [22]. The lower limit of normal (LLN) was used as a cut-off in the interpretation of respiratory function results.

An obstructive ventilatory defect (OVD) was defined by a FEV1/FVC ratio < LLN. A restrictive ventilatory defect (RVD) was established according to prebronchodilator spirometry as FEV1/FVC > 0.70 and a predicted FVC < LLN. A mixed pattern of obstruction and restriction was described as both a FEV1/FVC ratio and FVC < LLN.

14 female patients were unable to cooperate with spirometric testing. All of them showed no features suggestive of COPD (no exposure to risk factors for COPD, no medical history of chronic bronchitis, no wheezing/sibilants, and no X-ray finding of emphysema or chronic bronchitis) or neuromuscular/chest wall disorders and had no lung parenchymal abnormalities on chest X-ray.

#### 2.3.4. Sleep Polygraphic Study

A level three portable sleep polygraphic study including recording of oro-nasal flow (oro-nasal air pressure transducer), thoraco-abdominal movements (respiratory inductance plethysmography), body position, nocturnal oxygen saturation and heart pulse (pulse oximetry), and snoring (microphone placed on the anterior neck) was performed on all subjects. In patients without a known diagnosis of OSAHS before admission, sleep polygraphic studies were performed after hospital discharge with at least 6 weeks of clinical stability.

Respiratory events were scored manually by a sleep specialist according to the American Academy of Sleep Medicine (AASM) 2012 guidelines [23]. Obstructive sleep apnea was defined as a 90% decrease in airflow compared with baseline for at least 10 seconds, while there is evidence of persistent respiratory effort. Hypopnea was specified as a decrease in airflow amplitude by ≥30% of baseline, lasting for at least 10 seconds, and accompanied by oxygen desaturation ≥3%. OSAHS was diagnosed if the apnea hypopnea index (AHI) was ≥5 events/hour (h), with consistent clinical symptoms and/or comorbidities, or if AHI was ≥15 events/h with or without associated symptoms. OSAHS severity was graded according to AHI, as mild (5/h ≤ AHI < 15/h), moderate (15/h ≤ AHI < 30/h), or severe (AHI ≥ 30/h). Overnight pulse oximetry was analyzed to assess oxygen desaturation index per hour (ODI), mean overnight transcutaneous oxygen saturation (nSpO_2_), and total sleep time with nSpO_2_ which was less than 90% (TST90%).

#### 2.3.5. Echocardiographic Data

The following echocardiographic data were recorded: left ventricular ejection fraction (LVEF), left ventricular (LV) mass index, left ventricular diastolic function, and systolic pulmonary artery pressure (sPAP). Eccentric LV hypertrophy was defined by a LV mass index > 47 g/m^2^ with a relative wall thickness (RWT) of less than 0.45 [24]. Left ventricular diastolic function was assessed by mitral E/A ratio and tissue Doppler evaluation [25]. sPAP was estimated by the peak tricuspid regurgitant velocity. PHT was defined by an estimated sPAP > 35 mmHg, while a pressure > 45 mmHg was considered to indicate moderate to severe PHT [10].

### 2.4. Data Analysis

All the analyses were conducted using the Statistical Package for the Social Sciences version 20 software (SPSS, Chicago, IL). For quantitative variables, the Shapiro-Wilk test was used to check normal distribution, and descriptive characteristics were given as mean ± standard deviation (SD) when the distribution was normal and median with the interquartile range if the distribution was not normal. Categorical variables were expressed as the number of cases and percentages (%). Pearson's chi-square test (or Fisher exact test (when appropriate)) and Student *t*-test (or Mann–Whitney *U* test when indicated) were used to compare data between study groups. The Kaplan-Meier estimate of survival curve was used to determine the cumulative 1-year probability of hospital readmission and survival. A *p* value < 0.05 was considered statistically significant.

## 3. Results

### 3.1. Clinical Characteristics of Study Population

**Figure 1:**
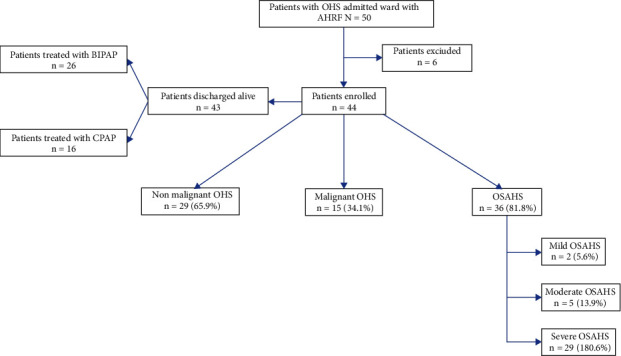
Composition of the study. OHS: obesity hypoventilation syndrome; OAHS: obstructive apnea-hypopnea syndrome; AHRF: acute hypercapnic respiratory failure; BIPAP: bilevel positive airway pressure; CPAP: continuous positive airway pressure; OSAHS: obstructive sleep apnea-hypopnea syndrome.

Of the 50 patients who met the inclusion criteria for study, 6 were excluded because of insufficient data in the medical record. Out of the 44 patients included, 15 (34.1%) and 29 (65.9%) were diagnosed with mOHS and non-mOHS, respectively. Thirty-six (81.8%) patients were classified as having OHS with coexisting OSAHS. Out of this last group, 2 patients (5.6%) had mild OSAHS, 5 patients (13.9%) had moderate OSAHS, and 29 patients (80.6%) had severe OSAHS. The patient's section is shown in Figure 1.

Three-quarters of patients (*n* = 33, 75%) were female. The patients' mean age was 66.5 ± 10.9 years. The mean admission BMI was 41.1 ± 6.8 kg/m^2^. The baseline demographic characteristics of the patients are summarized in Table 1.

**Figure 2:**
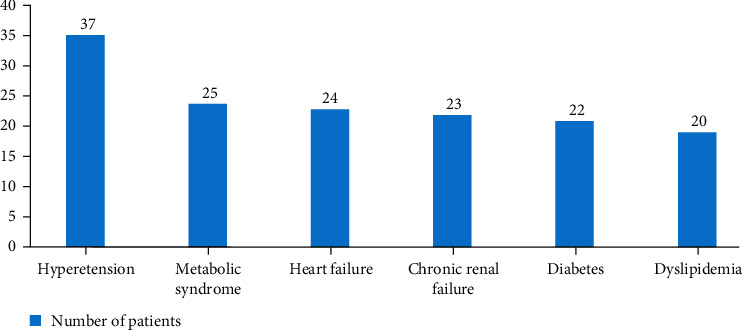
Cardiovascular and metabolic comorbidities in the study sample.

Recording of medical history showed an increased rate of cardiovascular comorbidities and risk factors (Figure 2). Major comorbidities seen in the overall sample were arterial hypertension (AHT) (*n* = 37; 84.1%), heart failure (*n* = 24; 54.5%), CRI (*n* = 23; 52.3%), diabetes (*n* = 22; 50%), dyslipidemia (*n* = 20; 45.5%), thromboembolism (*n* = 11, 25%), brain stroke (*n* = 5; 11.4%), and coronary artery disease (*n* = 5; 11.4%). Twenty-five (56.8%) patients met the adult treatment panel III (ATP-III) diagnostic criteria for metabolic syndrome. About half of the patients (*n* = 21, 47.7%) had a known history of OSAHS.

Almost one-fifth and a half of the patients had been hospitalized in the previous years, one or more times in an ICU or a pneumology department, respectively. The average number of admissions was 1 (range1-3). Eleven (25%) patients were on long-term CPAP therapy, and seven (15.9%) patients were on long-term oxygen therapy (LTOT), while 23 (52.3%) patients received long-term diuretic therapy (furosemide). Patients with mOHS had a significantly higher rate of heart failure (100% vs. 31%; *p* < 0.001), CRI (73.3% vs. 41.4%; *p* = 0.04), and dyslipidemia (66.7% vs. 34.5%; *p* = 0.04) than those with non-mOHS. The baseline medical history is outlined in Table 1.

More than two-thirds of patients (*n* = 30; 68.2%) were referred from the emergency department. Nearly one in nine patients (*n* = 5, 11.4%) were referred from the outpatients' clinic in our department, and one in 15 patients (*n* = 3; 6.8%) was transferred from an ICU.

At hospital admission, only 10 (22.7%) patients had been correctly diagnosed, while more than two-thirds (*n* = 34, 77.3%) were misdiagnosed as “asthma exacerbation” (*n* = 4, 4.9.1%), “COPD exacerbation” (*n* = 12, 27.3%), and/or heart failure (*n* = 29, 65.9%).

Acute cardiogenic pulmonary edema (ACPE) (*n* = 16, 36.4%), acute viral bronchitis (*n* = 12, 27.3%), and pneumonia (*n* = 5, 11.4%) were the main identified causal factors, while no cause had been specified in 5 (11.4%) patients.

### 3.2. Laboratory Data

The patients' pertinent laboratory data are presented in Table 2.

Over one-fifth of patients had high blood sugar levels (*n* = 10; 22.72%). Impaired creatinine clearance was identified in 36 (81.8%) patients. About one-third (*n* = 15, 34.1%) of patients had anemia, while hyperleukocytosis (*n* = 2; 4.5%) and thrombocytopenia (*n* = 4, 9.1%) were much less frequently observed. Transaminitis was present in more than one-fifth of patients (*n* = 10/42, 23.8%). Serum NT-pro BNP levels were measured in 30 patients and were increased in 10 (33.3%) patients. Among the 36 patients who had troponin measured, 14 (38.9%) had increased levels.

### 3.3. Respiratory Function Test

Spirometry data were available in 30 patients. All patients except 3 (*n* = 27/30) had RVD. One patient had a mild OVD, while the remaining two patients had no evidence of ventilatory defect. The median FEV_1_ was of 1.16 l (0.4-2.55), with a mean predicted percentage of 61% ± 20.7, the median FVC was of 1.39 l (0.48-3.84) with a mean predicted percentage of 59.5% ± 18.5, while the mean FEV1/FVC ratio was of 81.6% ± 7.

### 3.4. Echocardiography Data

Echocardiography was performed in 39 patients. The following echocardiographic data were obtained: LVEF of 60% (40–65%), with 6 (15.38%) patients having a LVEF ≤ 50%. All patients had LV hypertrophy, and the median mass index was 233 (145–408) g/m. Only 10 (25.6%) patients had echocardiographic features of LV diastolic dysfunction. The estimated median sPAP was 31 (20–75) mmHg. 18 (46.2%) patients had moderate-to-severe PHT. 19 (48.7%) patients had dilated right cavities.

### 3.5. Ventilatory Characteristics and NIPPV Related Side Effects

All patients (*n* = 44, 100%) were ventilated with BIPAP either at admission (*n* = 41, 93.2%) or after initial invasive ventilation (3 patients transferred from ICU). Setting for BIPAP over the first two days was an inspiratory positive airway pressure (IPAP) of 16.3 (12–25) and an expiratory positive airway pressure (EPAP) of 6 (4–9). Median overall duration was 9 hours per day (1.3–20). Ventilator therapy duration was significantly longer in patients with mOHS than in those with non-mOHS (*p* = 0.01). Four main side effects of ventilation therapy were detected: eye irritation (*n* = 11, 25%), nasal irritation (*n* = 10, 22.7%), mouth dryness (*n* = 10, 22.7%), and skin lesions (*n* = 7, 15.9%).

### 3.6. Outcomes and Follow-Up of Patients

**Figure 3:**
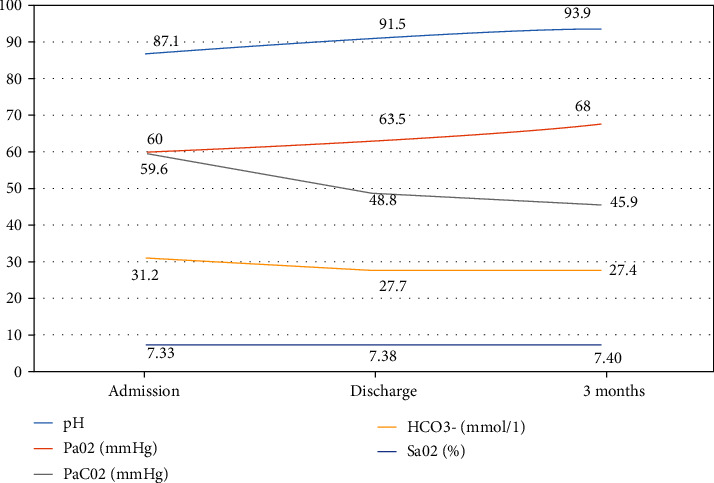
Arterial blood gases at the time of noninvasive positive pressure ventilation, at hospital discharge, and at 3 months follow-up after hospital discharge. PaCO_2_: arterial partial pressure of carbon dioxide; PaO_2_: arterial partial pressure of oxygen; HCO3: concentration of bicarbonate in arterial blood; SaO_2_: oxyhemoglobin saturation of arterial blood gas.

BIPAP therapy failed in one patient (2.27%), leading to intubation and invasive mechanical ventilation. ABG measurements at hospital discharge and 3-month follow-up were similar. However, they were significantly improved when compared with measurements at patient's admission (Figure 3).

**Figure 4:**
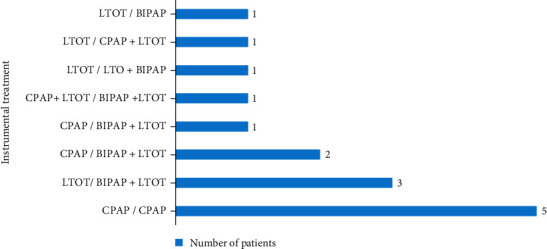
Instrumental respiratory therapy before admission for AHRF and after hospital discharge. LTOT: long-term oxygen therapy; CPAP: continuous positive airway pressure; BiPAP: bi-level positive airway pressure.

Of the 43 patients discharged alive, only one patient did not receive instrumental respiratory therapy. Domiciliary BIPAP was used in 26 patients (61.9%), 4 patients of whom were already ventilated with CPAP prior to admission. Nocturnal CPAP was used in 16 patients (38.1%), including 7 patients who were already under CPAP before admission. LTOT was used in one-fifth of patients (*n* = 9, 21.4%), combined with CPAP in 2 patients (4.8%), and with BIPAP in 7 other patients (16.7%) (Figure 4). No significant difference was found in instrumental respiratory therapy at discharge, either between men and women or between patients with mOHS and those with non-mOHS (Table 3). Similarly, age, BMI, overall daily NIV duration, and idiopathic cause did not differ significantly between patients ventilated with BIPAP and those treated with CPAP (Table 4).

**Figure 5:**
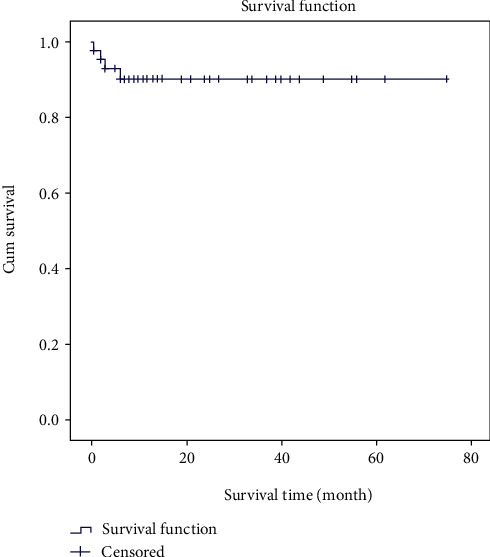
Survival of patients during the study follow-up period.

In total, four cases of death had been reported, one of which happened during the index hospitalization. The three others occurred at 2, 3, and 6 months after discharge from the hospital. The probability of survival was about 90% at 6 and 12 months (Figure 5).

**Figure 6:**
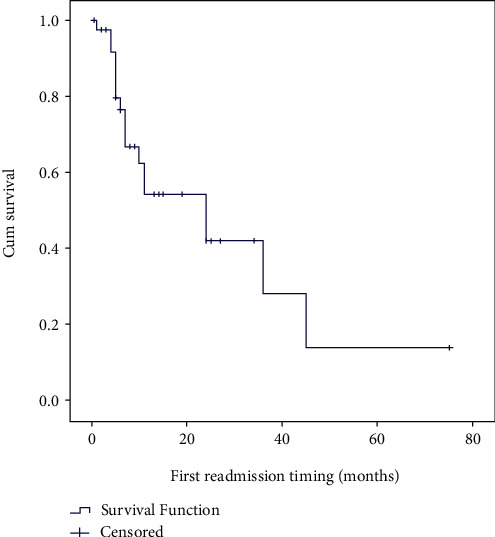
Probability of remaining free from hospital readmission for a new episode of AHRF during the study follow-up period.

Of the 43 patients discharged alive from the hospital, 10 patients were lost to follow-up. The median follow-up duration was 13 months [1-75]. More than a third of patients (*n* = 18, 41.9%) had been readmitted one or more times (range from 1 to 6 times) with a new episode of AHRF, with a median readmission time interval of 7 months (range from 1 to 45 months). The probability of readmission was 56% at 6 months and 22% at 12 months, respectively (Figure 6). Age, gender, severity of OHS, BMI, CVF, and the type of home NIPPV had no significant influence on the probability of readmission for a new episode of AHRF.

## 4. Discussion

### 4.1. Major Findings

This is a retrospective observational cohort study conducted in Tunisia and investigating obese patients with AHRF. In most if not all of them, it was an acute on CRF, as suggested by the high serum bicarbonate levels at admission (Table 2). We found that OHS was still a frequently unrecognized cause of AHRF, that ACPE and viral bronchitis were the two main precipitating factors of AHRF in patients with OHS, and that BIPAP was a highly safe and effective management option of AHRF in patients with OHS. Moreover, patients treated with home NIPPV after an AHRPF episode had a good outcome with a low long-term mortality rate. Finally, the risk of readmission for a new episode of AHRF did not differ between BIPAP and CPAP therapy.

Despite the increasing obesity prevalence in the Tunisian adult population, only 44 episodes of AHRF were linked with OHS over the most recent 13-year study period. In no case, OHS had been previously diagnosed, although 1 in 5 patients and half of the patients had been previously hospitalized one or more times in the ICU or the pulmonology department, respectively. This fact suggests that OHS remains widely unknown even among specialists. Thus, it is frequently missed or neglected during hospitalization as well as during outpatient follow-up after hospital discharge [26]. Moreover, more than two-thirds of patients had been erroneously diagnosed on admission with COPD/asthma exacerbation and/or congestive heart failure. Yet, none of the patients diagnosed with COPD had evidence of obstructive airway disease, and none of the patients diagnosed with asthma had evidence of reversible airway obstruction on spirometry. Similarly, in a study by Akpinar [27], of a total of 82 patients hospitalized with AHRF, none was diagnosed with OHS. These data indicate that OHS often remains unrecognized until an episode of HARF occurs. It is also a still unrecognized cause for AHRF. In the study of Marik and Desai [10], of 51 patients with severe OHS admitted with AHRF, only 3 patients had been previously diagnosed with OHS. In another investigation including 53 patients, Bry et al. [28] reported that the diagnosis of OHS was made prior to the AHRF episode in only 8 patients. This is despite the fact that obesity associated with hypoventilation has been found to be frequent among hospitalized patients. In a research by Nowbar et al. [29], hypoventilation (mean PaCO_2_ of 52 ± 7 mmHg) was present in 31% (*n* = 47) of 52 hospitalized patients with severe obesity (IMC ≥ 35 kg/m^2^) who did not have other reasons for hypercapnia.

The underdiagnosis of OHS leads to the fact that most patients are diagnosticated late, after the occurrence of detrimental outcomes including mainly AHRF, severe multisystem diseases directly linked with obesity, chronic respiratory failure, and/or chronic hypoventilation. Carrillo et al. [30] studied 173 patients with OHS hospitalized with AHRF. Their patients were older (74 ± 11 vs. 66.5 + 10.9, respectively), but were as obese as the patients in our cohort (BMI = 42 ± 6 vs. 41 ± 6.8 kg/m^2^, respectively). Moreover, they were for the most part women as it was observed in our study (76.45% vs. 75%, respectively). In an analysis by Bry et al. [28], patients were younger (61 ± 10 vs. 66.5 ± 10.9, respectively), female predominance was less important (50% vs. 75%, respectively). In contrast, BMI (42 ± 11 vs. 41.1 ± 6.8, respectively) was similar to our data. Interestingly, our review also differs from the two abovementioned studies [28] [30] for a higher level of comorbidities, mainly cardiovascular and metabolic disorders. This finding could be explained by the fact that half of our patients (*n* = 20, 50%) had morbid obesity (IMC ≥ 40 kg/m^2^), including a group of 15 patients (34.1%) with mOHS. Actually, this group was found to have significantly higher rates of heart failure (100% vs. 31%; *p* < 0.001), CRI (73.3% vs. 41.4%; *p* = 0.04), and dyslipidemia (66.7% vs. 34.5%; *p* = 0.04) compared to the group of patients with non-mOHS.

Morbid obesity is commonly associated with various multivisceral complications such as respiratory failure, PHT, OSAH, complicated diabetes, hypertrophic cardiomyopathy, metabolic syndrome, vitamin D deficiency, and muscular deconditioning (M. [31]). Based on this, a new concept called “mOHS” has been recently defined by Marik as a subgroup of OHS in which morbid obesity (BMI > 40 kg/m^2^) is associated with increased multiorgan system dysfunction and morbidity [10]. Older patients with mOHS are commonly fragile in the medical sense of the term, bedridden, over dependent, and with a poor prognosis. Therefore, most of these patients are classified as “do not intubate.” In a prospective study by Lemyze et al. [32], of 73 morbidly obese patients admitted in ICU with AHRF, 60% were concerned by a “do not intubate order.” This emphasizes the importance of correct identification of patients with mOHS to ensure appropriate management and to avoid futile therapeutic escalation [31]. The largest study of patients with mOHS ever published was by Marik and Desai [10]. The 61 patients included in this study had severe multisystem diseases directly associated with morbid obesity. In contrast to these results, our study showed no significative differences in baseline characteristics between mOHS and nmHOS, apart from BMI and AHI (which was lower in the malignant form). Besides, both groups received similar respiratory therapy at discharge and had comparable long-term outcomes. These findings based on a too small sample seem to be misleading and should not be extrapoled to other populations.

Obstructive sleep apnea associated with alveolar hypoventilation is the most common ventilator pattern observed in obese patients hospitalized with AHRF [33]. In our study, the majority of patients (*n* = 34; 81.1%) had OSAHS confirmed by sleep polygraph recording: 21 patients had a previous known history of OSAHS while the other 15 patients were diagnosed with OSAHS after the index event of AHRF. Cuvelier et al. [33] studied 20 obese patients hospitalized with AHRF. The sleep recording performed one month after hospitalization showed OSAHS in 9 patients, whereas 11 other patients were diagnosed with pure OHS. Rabec et al. [34] reported results based on a larger sample composed of 41 obese patients hospitalized with AHRF and treated with NIPPV. Among these, 6 patients had OSAHS, 19 had OHS (associated with OSAHS in 16 cases), 4 had COPD, and 10 were diagnosed with overlap syndrome (combination of COPD and OSAH). These results confirm the fact that OSAHS is highly prevalent in obese patients hospitalized with AHRF. Unfortunately, most of these patients did not have a respiratory polygraphy at baseline and could not undergo polygraphy during episodes of AHRF. Therefore, for many of them, the presence of OSAHS could only be strongly suspected until confirmation of the diagnosis far from the acute episode of respiratory failure [28]. Nevertheless, we think that each patient admitted with AHRF should be considered at high risk for OSAH and should undergo respiratory polygraphy after this acute event, unless the diagnosis of OSAHS was already known prior to admission.

In our study, the following echocardiographic abnormalities were identified: LV hypertrophy in all patients (100%), moderate-to-severe PHT and dilated right cavities each one in almost half of patients (46.2% and 48.7%, respectively), LV diastolic dysfunction in about one quarter of patients (25.6%), and a LV systolic dysfunction (LVEF ≤ 50%) in 13.63% of patients. In the study of Carrillo et al. [30], two main cardiac abnormalities were reported: right ventricle dilatation (*n* = 16, 23%) and left ventricle diastolic dysfunction (*n* = 19, 28%). Marik and Desai [10] described higher rates of cardiac abnormalities in patients with mOHS as 71% of them had LV hypertrophy, 61% had LV diastolic dysfunction, and 77% had HTP. The impaired cardiac function commonly observed in OHS patients is closely associated with several comorbid conditions mainly systemic AHT, diabetes, dyslipidemia, and obesity. Obesity has been identified as a strong and independent risk factor for LV hypertrophy and cardiac dysfunction. Both the high cardiac output and excessive production of proinflammatory adipokines associated with obesity contribute to the development of LV hypertrophy in obese patients. The chronic hypoxemia associated with OHS may also be involved as suggested by Sugerman et al. [35].

The mean FVC in our patients was of 59.5% ±18.5 of the predicted value, lower than what is usually reported in stable patients with OHS [36]. Similarly, in a study by Chebib et al. [26], patients with OHS who were admitted to the ICU for AHRF had significantly lower baseline FVC than those not admitted to the ICU (72% vs. 80%, respectively; *p* = 0.01). Moreover, a cut-off of FVC < 3.5 L in men and 2.3 l in women could predict chronic daytime hypercapnia in obese subjects, as was shown by Mandal et al. [37]. These findings suggest that a severe restrictive spirometric pattern might be considered as a risk factor for AHRF in patients with OHS [38].

More than a third of our patients were recorded as having a period between symptom onset and hospitalization equal to or greater than 15 days. This long prehospital delay time indicates that AHRF in OHS patients may occur either acutely or insidiously with a progressive deterioration in gas exchange [39]. Besides, clinical presentation with hypercapnic encephalopathy syndrome (HES) may be underestimated or misdiagnosed, resulting in a delayed consultation. HES is a heterogeneous and potentially reversible wide range of neurological alterations (headache, ataxia, cognitive defects, psychomotor agitation, confusion, flapping, tremor, daytime sleepiness, delirium, and coma) occurring as a result of severe decompensated respiratory hypercapnic acidosis [40] [41]. In our study, daytime sleepiness, headaches, and behaviour disturbance were reported by about two-thirds, half, and the quarter of patients, respectively. One patient who presented at the emergency with behaviour disturbance was initially suspected of having a psychological or mental disorder. These findings corroborate the fact that HES is common in patients with severe AHRF. It may also be the dominant clinical feature of severe AHRF, mimicking cognitive, and/or neuropsychological disorders and leading to inappropriate or delayed diagnosis and treatment.

In our study, respiratory infection (*n* = 17; 38.63%) and ACPE (*n* = 12; 36.4%) were the two main precipitating factors of AHRF. In contrast, pleural effusion (*n* = 2, %) and extra respiratory infection (*n* = 1; %) were rarely involved. In a study by Chebib et al. [26], congestive heart failure was responsible for AHRF in more than half of patients (54%), followed by acute pulmonary embolism (10.8%), and in slightly less than one-third of patients, no cause could be identified. Carrillo et al. [30] found that most of AHRF episodes were triggered by respiratory infections, while cardiac origin was much less involved than was reported in our study. For a minority of patients, no cause could be determined as was the case for our patients (6% vs. 11.4%, respectively). In an analysis of Bry et al. [28], three main origins were identified: respiratory tract infections (32%), ACPE (28%), and exacerbation of COPD. However, most of the reported AHRF cases were regarded as idiopathic (62%) contrary to our results and those of Carrillo et al. [30]. Similarly, causes of AHRF were specified in only 21 among 61 patients (34.42%) assessed by Marik and Desai [10]. Identified causes in this study were dominated by pneumonia, extra respiratory infections (urosepsis and limb cellulitis), and acute CRI. Overall, the common causes of AHRF in patients with OHS are respiratory infections, ACPE, and sepsis [42], but idiopathic forms are also frequent. Therefore, OHS patients with AHRF need to be meticulously assessed for infection, with a careful examination of heart function and loading condition [26]. Other less common causes include pleural effusion, pulmonary embolism, depressants, surgery, rib contusion and fractures, supine immobilization, and thoracic bracing [43] [30] [28].

In our cohort, NIPPV using BIPAP was very highly effective to treat AHRF, with only 2.27% of patients failing the modality. Moreover, NIPPV was well tolerated by most of the patients, as no serious side effects or complications were observed. In cohorts of OHS patients admitted with AHRF, the efficacy of NIPPV was highly variable, with reported failure rates of between 0% and 60% (De [44]). According to these studies, higher NIPPV success rates were observed in OHS patients with idiopathic episodes of AHRF and those with high PaCO_2_ levels [28] [26] [30] [45]. In contrast, higher NIPPV failure rates were found to be associated with several factors including mainly mOHS, severe hypoxemia, and infectious pneumonia as the main precipitating factor of AHRF (Malcolm [32]) [10][45] [46]. Both Lemyze et al. [32] and Marik and Desai [10] reported high NIPPV failure rates of 17% and 39.65%, respectively, in severely obese patients (mean BMI of 46.6 and 48.9 kg/m^2^, respectively) with mOHS. Of interest, initial IPAP and EPAP levels were higher in our study than in the study by Lemyze et al., which may have helped us achieve a higher success rate, this is despite a high prevalence of mOHS (more than one-third of our patients), and a small nurse-to-patient ratio. As was mentioned in a review by Shah et al. [47], high EPAP and IPAP levels are usually required to successfully ventilate patients with OHS. Initial EPAP levels should be set at least at 5 cmH_2_O, but a maximum of 15 cmH_2_O may be required particularity during sleep. IPAP should be at least 10 cmH_2_O higher than EPAP and can be as high as 30 cmH_2_O (De [44]). Our study corroborates data from many other studies supporting the efficacy of NIPPV in the management of AHRF in patients with OHS. Even better, Carrillo et al. [30] found that NIPPV tended to be more effective in OHS patients than in COPD patients, with better outcomes including lower rates of heart failure, lower intrahospital mortality, and a higher survival rate at 1 year. Despite all these data, current guidelines recommend the use of NIPPV in AHRF in COPD patients. In contrast, there is not yet a published consensus on the indications for NIPPV in AHRF complicating OHS, although new guidelines were recently published for the management of stable OHS [14].

Of our 43 patients discharged alive, domiciliary NIPPV was used in 26 patients (61.9%), while nocturnal CPAP was used in 16 patients (38.1%). Taking as reference point the results at admission, we observed an improvement in gas exchange as was attested by the significant improvement in ABG measurements at 3 months after in-hospital initiation of NIPPV therapy. However, this improvement was not significant when compared with the results at hospital discharge. Moreover, no significant difference was found in ABG measurements at 3 months between BIPAP and CPAP therapy. Global readmission rate for ARF was 56% at 6 months and 22% at 1 year and was independent of sex, BMI, OHS severity, and of domiciliary NIPPV modality (BIPAP or CPAP). While COPD is well known for its association with a high rate of readmission after an episode of AHRF treated with NIPPV (ranging between 56% and 80% at 1 year) [48] [49], few data are available regarding patients with OHS. Chebib et al. [26] reported that 46% of patients with OHS admitted to the ICU for AHRF were readmitted for the same reasons in the following 2 years. In a study by Bry et al. [28], the global readmission rate for AHRF at 1 year was 20%. Patients ventilated at home had lower 1-year readmission rates than those who were not; however, the difference was not significant (10% vs. 36%, respectively; *p* = 0.07). Carrillo et al. [30] found that patients with OHS had a 1-year readmission rate as high as that observed in patients with COPD (>50%). Curiously, patients with OHS treated with domiciliary NIPPV had a higher 1-year readmission rates when compared to those who were not ventilated at home. Yet, this difference did not remain significant after adjustment for confounding variables (adjusted OR 1.31; 95% CI, 0.71-2.41; *p* = 0.39). Taken together, these data suggest that readmission to hospital after an episode of AHRF treated by NIPPV is probably more frequent in patients with COPD than in patients with OHS. However, they do not allow to conclude as to the type of home ventilation and the modality of ventilation that could reduce or not the long-term readmission probability in the latter.

Our study had several limitations. First, given the retrospective nature, not all potentially eligible patients could be included either because of lack of data or undiagnosed cases of OHS. This led to a small sample size, with many patients lost to follow-up. Some key variables such as the impact of the interface on the effectiveness of NIPPV therapy, home NIPPV compliance could not be assessed. Moreover, we could not be aware about deaths occurring out hospital in patients lost to follow-up, which would imply an underestimation in mortality rate. Second, the baseline ventilator patterns (pure OHS, pure OSAHS, or OHS + OSAHS) were not known at admission of patients, which may have impacted the titration of BIPAP setting. If we add to that the monocentric design of the study, our results may not be reproducible in other centers with different approaches to BIPAP and different inpatient hospitals. The limited number of patients with the small number of events (only 1 BIPAP failure event out 44 patients with OHS and 4 deaths) do not allow any multivariate analysis of our results [50]. Finally, the observational design of our study did not allow clear conclusions about the efficiency of BIPAP in the AHRF management; therefore, controlled randomized trials are still required.

## 5. Conclusion

Our study shows that OHS remains a frequently unrecognized cause of AHRF even in morbidly obese patients. The two leading precipitating factors of AHRF in patients with OHS are ACPE and respiratory infections, hence, the need for a careful examination of heart function and loading condition, with appropriate investigations for infections. BIPAP is highly effective in the management of AHRF even in patients with mOHS. All patients discharged alive except one were treated with home NIPPV, either BIPAP or CPAP, and had a low long-term mortality rate. No factor is independently associated with a higher risk for readmission.

## Figures and Tables

**Table 1:** Clinical, functional, and therapeutic characteristics of patients.

	Total sample (*n* = 44)	Nm-OHS (*n* = 29)	M-OHS (*n* = 15)	*p*
Age	66.5 (10.9)^∗^	64.8 (9.6)^∗^	69.6 (12.7)^∗^	0.17
Sex, M/F *n* (%)	11(25)/33(75)	7(24.1)/22(75.9)	4(26.7)/11(73.3)	1
BMI, kg/m^2^	41.1 (6.8)^∗^	37.5 (30–60)^∗∗^	45 (40–50)^∗∗^	<0.001
Active or ex smoking, *n* (%)	12 (27.3)	7 (24.1)	5 (33.3)	0.72
Charlson comorbidity score (median, IQR)	4 (1–10)	4 (1–9)	4.5 (1–10)	0.33
Previous LTOT, *n* (%)	7 (15.9)	5 (17.2)	2 (13.3)	1
Previous home CPAP, *n* (%)	11 (25)	7 (24.1)	4 (26.7)	1
Previous home BIPAP, *n* (%)	0			
Previous ICU hospitalization, *n* (%)	11 (25)	7 (24.1)	4 (26.7)	1
Previous ward hospitalization, *n* (%)	23 (52.3)	14 (48.3)	9 (60)	0.46
*Respiratory tests*				
FEV1				
Ml (median, IQR)	1160 (400–2550)	1170 (400–2550)	1080 (760–2330)	0.98
% pred	61 (20.7)^∗^	61 (20–122)^∗∗^	73 (27–94)^∗∗^	0.13
FVC				
Ml (median, IQR)	1390 (480–3840)	1390 (480–3840)	1160 (1000–2890)	0.93
% pred (mean, SD)	59.6 (18.6)	59.1 (19.4)	60.8 (18.1)	0.87
FEV1/FVC, % (mean, SD)	81.6 (7)	80.4 (6.7)	83.8 (7.3)	0.2
*Cardiac function*				
LVEF, % (median, IQR)	60 (40–65)	60 (40–65)	61 (48–65)	0.22
LV diastolic dysfunction, *n* (%)	10 (25.6)	8 (32)	2 (14.3)	0.28
LV mass index, g/m (median, IQR)	233 (145–408)	233 (155–408)	251 (145–380)	0.33
sPAP, mmHg (median, IQR)	31 (20–75)	32 (20–60)	31 (20–75)	0.89
Dilated right heart cavities, *n* (%)	19 (48.7)	13 (52)	6 (42.9)	0.58
*Overnight type 3 PSG data*				
AHI, h^−1^	48.6 (9–125.7)^∗∗^	56.9 (30.6)^∗^	50.5 (37.2)^∗^	0.59
T90%, (median, IQR)	85.2 (6.5–99.8)	85.2 (6.5–99.5)	87.8 (42–99.8)	0.97
*Origin,n(%)*				
Emergency room	30 (68.2)	22 (75.9)	8 (53.3)	0.17
Ward	5 (11.4)	2 (6.9)	3 (20)	0.32
ICU	3 (6.8)	1 (3.4)	2 (13.3)	0.26
*AHRF cause,n(%)*				
APE	16 (36.4)	10 (34.5)	6 (40)	0.72
Viral bronchitis	12 (27.3)	8 (27.6)	4 (26.7)	1
Pneumonia	5 (11.4)	3 (10.3)	2 (13.3)	1
Other	11 (25)	8 (27.6)	3 (20)	1
*Ventilatory treatment during AHRF*				
IPAP, cmH_2_O (median, IQR)	16.3 (12–25)	17 (12–24)	15 (14–25)	0.31
EPAP, cmH_2_O (median, IQR)	6 (4–9)	6 (4–9)	6 (4–8)	0.53
Duration, h/day (median, IQR)	9 (1.3–20)	8 (1.3–20)	10 (6–18)	0.01
Failure rate, *n* (%)	1 (2.3)	1 (3.4)	0 (0)	1
Length of hospital stay, days (median, SIQ)	20.5 (3–90)	22 (3–90)	18 (10–66)	0.35
*Home treatment after discharge,n(%)*				
CPAP	16 (38.1)	11 (39.3)	5 (35.7)	0.82
BIPAP	26 (61.9)	17 (60.7)	9 (64.3)	0.82
LTOT	9 (21.4)	7 (25)	2 (14.3)	0.69

Nm-OHS: nonmalignant obesity hypoventilation syndrome; M-OHS: malignant OHS; *p*: *p* value; M: male; F: female; LTOT: long-term oxygen therapy; CPAP: continuous positive airway pressure; BIPAP: bilevel positive airway pressure; ICU: intensive care unit; FEV1: forced expiratory volume first second; FVC: forced vital capacity; Pred: predicted; LVEF: left ventricular ejection fraction; LV: left ventricular; sPAP: systolic pulmonary artery pressure; AHI: apnea hypopnea index; TST90%: total sleep time with nSpO_2_ was less than 90%; AHRF: acute hypercapnic respiratory failure; APE: acute pulmonary oedema; IPAP: inspiratory positive airway pressure; EPAP: expiratory positive airway pressure. ^∗^: mean (standard deviation). ^∗∗^: median (interquartile range). BMI: body mass index.

**Table 2:** Laboratory data of patients at admission.

	Specimens *n*, %	Total sample (*n* = 44)	Nm-OHS (*n* = 29)	M-OHS (*n* = 15)	*p*
Glucose serum level, mmol/l (median, IQR)	43 (97.7)	6.9 (2.7–27.9)	6.9 (2.7–24)	6.8 (4.7–27.9)	0.65
BUN, mmol/l (median, IQR)	43 (97.7)	8.4 (2.7–35)	8.7 (2.7–19.6)	8.4 (4.2–35)	0.89
Serum creatinine level, *μ*mol/l (median, IQR)	44 (100)	89.5 (42.7–281)	85 (42.7–183)	93 (47–281)	0.24
Creatinine clearance, ml/min (mean, SD)	44 (100)	67.1 (29.4)	69.9 (27.9)	61.6 (32.5)	0.38
AST, U/l (median, IQR)	42 (95.5)	15.5 (2–67)	16.5 (2–44)	14 (8–67)	0.94
ALT, U/l (median, IQR)	42 (95.5)	16 (6–49)	16 (6–49)	17 (6–42)	0.63
HB, g/dl (median, IQR)	44 (100)	12.9 (6.6–21.2)	13.4 (6.6–21.2)	12.5 (10.7–16.1)	0.52
Hematocrit, %	44 (100)	41.5 (30.7–65)^∗∗^	42.7 (7.3)^∗^	41.6 (4.4)^∗^	0.57
PLQ, e/ml (mean, SD)	44 (100)	228.84 (60.157)	226.48 (66.180)	233.40 (48.186)	0.72
WBC, e/ml (mean, SD)	44 (100)	8.911 (2.502)	8.596 (2.762)	9.520 (1.837)	0.25
Troponin, *μ*g/L (median, IQR)	36 (81.8)	0.03 (0–64)	0.02 (0–0.54)	0.04 (0.0–64)	0.4
Pro-BNP, pg/mL (median, IQR)	30 (68.2)	267 (18–7414)	135 (18–2524)	500 (30.3–7414)	0.2
CRP, mg/L (median, IQR)	40 (90.9)	9.5 (2–356)	9 (2–62)	16.9 (5–356)	0.48
TSH, mUI/L (median, IQR)	23 (52.3)	2.1 (0.25–9.95)	1.82 (0.45–5.66)	2.11 (0.25–9.95)	0.78
Serum protein, g/l (mean, SD)	22 (50)	72.7 (6.2)	74.3 (6.3)	70.3 (5.6)	0.15
pH (median, IQR)	44 (100)	7.33 (7.20–7.35)	7.33 (7.20–7.35)	7.33 (7.27–7.35)	0.5
PaCO2, mmHg (median, IQR)	44 (100)	59.6 (46–97.2)	59 (48.5–97.2)	61 (46–86.3)	0.59
PaO2, mmHg (median, IQR)	44 (100)	60 (34–149)	60 (34–90)	61 (40–149)	0.98
HCO_3_^−^, mmol/l (mean, SD)	44 (100)	32 (5.1)	31.8 (5)	32.2 (5.4)	0.83
SaO_2_, % (median, IQR)	44 (100)	87.1 (55.7–99)	86.3 (55.7–99)	87.9 (67–99)	0.79

Nm-OHS: nonmalignant obesity hypoventilation syndrome; M-OHS: malignant OHS; *p*: *p* value; IQR: interquartile range; SD: standard deviation; BUN: blood urea nitrogen; AST: aspartate aminotransferase; ALT: alanine aminotransferases; HB: hemoglobin; PLQ: platelet count; WBC: white blood cells; Pro-BNP: probrain natriuretic peptide; CRP: C-reactive protein; TSH: thyroid-stimulating hormone. ^∗^: mean (standard deviation). ^∗∗^: median (interquartile range). PaCO_2_: arterial partial pressure of carbon dioxide; PaO_2_: arterial partial pressure of oxygen; HCO3-: concentration of bicarbonate in arterial blood; SaO_2_: oxyhemoglobin saturation of arterial blood gas.

**Table 3:** Discharge instrumental respiratory therapy in the overall population, in men, in women, in patients with malignant OHS, and patients with nonmalignant OHS.

	Total (*n* = 42)	Male (*n* = 10)	Female (*n* = 32)	*p*	Nonmalignant OHS (*n* = 28)	Malignant OHS (*n* = 14)	*p*
LTOT *n* (%)	9 (21.4)	0 (0)	9 (28.1)	0.09	7 (25)	2 (14.3)	0.69
CPAP *n* (%)	16 (38.1)	4 (40)	12 (37.5)	1	11 (39.3)	5 (35.7)	0.82
CPAP + LTOT *n*, (%)	2 (4.8)	0 (0)	2 (6.3)	1	2 (7.1)	0 (0)	0.54
BiPAP *n*, (%)	26 (61.9)	6 (60)	20 (62.5)	1	17 (60.7)	9 (64.3)	0.82
BiPAP+ LTOT *n*, (%)	7 (16.7)	0 (0)	7 (21.9)	0.17	5 (17.9)	2 (14.3)	1

*p*: *p* value; OHS: obesity hypoventilation syndrome; LTOT: long-term oxygen therapy; CPAP: continuous positive airway pressure; BiPAP: bilevel positive airway pressure.

**Table 4:** Association between discharge instrumental respiratory therapy and age, BMI, and overall daily NIPPV duration.

	CPAP (*n* = 16)	BiPAP (*n* = 26)	*p*
Age (years) mean, (SD)	63.4 (9.8)	67.4 (11.1)	0.25
BMI (kg/m^2^) mean, (SD)	41.3 (7.8)	41 (6.5)	0.9
Idiopathic cause *n*, (%)	2 (12.5)	3 (11.5)	1
Overall daily NIPPV duration median, (IQR)	8 (1.3–16)	9 (2–20)	0.13

BMI: body mass index; CPAP: continuous positive airway pressure; BiPAP: bilevel positive airway pressure; *p*: *p* value; SD: standard deviation; NIPPV: noninvasive positive pressure ventilation; IQR: interquartile range.

## Data Availability

Data are available on request through the authors.
